# Five-Year Risk Prediction Models for Peripheral Artery Disease in Patients With Chronic Kidney Disease

**DOI:** 10.1016/j.xkme.2026.101351

**Published:** 2026-04-03

**Authors:** Jing Chen, Ling Tian, Joshua D. Bundy, Byron C. Jaeger, Ruiyuan Zhang, Changwei Li, Hua He, Damodar Kumbala, Chung-Shiuan Chen, Pranav S. Garimella, Raymond R. Townsend, Mahboob Rahman, Bernard G. Jaar, Stephen M. Sozio, Madhumita Jena Mohanty, Jason Roy, Xiaoming Zhang, Esteban Cedillo-Couvert, Edward Horwitz, Matthew J. Budoff, Kunihiro Matsushita, Harold I. Feldman, L. Lee Hamm, Jiang He, CRIC Study Investigators, Amanda H. Anderson, Lawrence J. Appel, Debbie L. Cohen, Laura M. Dember, Alan S. Go, James P. Lash, Mahboob Rahman, Panduranga S. Rao, Vallabh Shah, Mark L. Unruh

**Affiliations:** 1 Department of Internal Medicine, UT Southwestern Medical Center, Dallas, TX; 2 Department of Epidemiology, UT Southwestern Medical Center, Dallas, TX; 3 The Charles and Jane Pak Center for Mineral Metabolism and Clinical Research, UT Southwestern Medical Center, Dallas, TX; 4 Division of Cardiology, Department of Medicine, Northwestern University Feinberg School of Medicine, Chicago, IL; 5 Department of Epidemiology, Tulane University School of Public Health and Tropical Medicine, New Orleans, LA; 6 Division of Public Health Science, Wake Forest University School of Medicine, Winston-Salem, NC; 7 Renal Associates of Baton Rouge, Baton Rouge, LA; 8 Department of Medicine, University of California San Diego School of Medicine, San Diego, CA; 9 Department of Medicine, University of Pennsylvania Perelman School of Medicine, Philadelphia, PA; 10 Department of Medicine, Case Western Reserve University School of Medicine, Cleveland, OH; 11 Department of Medicine, Johns Hopkins University School of Medicine, Baltimore, MD; 12 Department of Epidemiology, Johns Hopkins Bloomberg School of Public Health, Baltimore, MD; 13 Department of Medicine, Wayne State University, Detroit, MI; 14 Department of Biostatistics and Epidemiology, Rutgers University School of Public Health, Piscataway, NJ; 15 Department of Biostatistics, Epidemiology and Informatics, University of Pennsylvania Perelman School of Medicine, Philadelphia, PA; 16 Department of Medicine, University of Illinois at Chicago College of Medicine, Chicago, IL; 17 MetroHealth Medical Center, Cleveland, OH; 18 The Lundquist Institute at Harbor-UCLA Medical Center, Division of Cardiology, Los Angeles, CA; 19 Department of Medicine, Tulane University School of Medicine, New Orleans, LA

**Keywords:** Ankle-brachial index, chronic kidney disease, peripheral artery disease, risk factors, risk prediction models

## Abstract

**Rationale & Objective:**

Patients with chronic kidney disease (CKD) have an increased risk of peripheral artery disease (PAD), yet no PAD risk-prediction models currently exist for this population. We developed and internally validated 5-year PAD risk-prediction models for individuals with CKD.

**Study Design:**

Prospective cohort study.

**Setting & Participants:**

3,076 patients with CKD without PAD from the Chronic Renal Insufficiency Cohort (CRIC) study.

**Exposure:**

Clinically available variables, ankle-brachial index (ABI), and non-routinely measured cardiovascular disease biomarkers assessed at baseline.

**Outcomes:**

New-onset adjudicated clinical PAD event or an ABI ≤0.9 at an annual follow-up visit.

**Analytical Approach:**

Cox proportional hazards models were applied to estimate 5-year risk of incident PAD from baseline. Model performance was assessed by discrimination, calibration, and net reclassification improvement. All models were internally validated using Monte Carlo cross-validation.

**Results:**

Participants had a mean age of 57 years; 55% were male and 40% were Black. Over 5 years, 512 developed PAD. Compared with an ABI-only model that included ABI, age, and sex (area under the receiver operating characteristic curve [AUC], 0.697; 95% CI, 0.688-0.713), a clinical model using routine variables showed similar discrimination (AUC, 0.682; 95% CI, 0.669-0.691; *P* = 0.09). Adding ABI to the clinical model improved discrimination (AUC, 0.721; 95% CI, 0.709-0.736; *P* <0.001). The biomarker-enhanced model achieved an AUC of 0.724 (95% CI, 0.711-0.745), which was not significantly different from the clinical model with ABI. Both the clinical model with ABI and the biomarker-enhanced model were well calibrated and improved reclassification of non-events compared with the ABI model (19.7%; 95% CI, 17.5-22.0% and 22.2%; 95% CI, 19.8-24.5%, respectively).

**Limitations:**

Lack of external validation.

**Conclusions:**

A PAD risk prediction model that combines clinical variables with ABI improves the identification of patients with CKD at high risk for PAD better than ABI alone.

## Plain-Language Summary

Patients with chronic kidney disease (CKD) are at increased risk of peripheral artery disease (PAD), yet no risk prediction models currently exist for this population. We developed and internally validated models for 5-year PAD risk in CKD. An ankle-brachial index (ABI) model (ABI, age, sex) and a clinical model that included 12 readily available variables (age, sex, smoking status, cardiovascular disease, hemoglobin A_1c_, pulse pressure, antihypertensive use, low-density lipoprotein cholesterol, high-density lipoprotein cholesterol, alkaline phosphatase, parathyroid hormone, and bicarbonate) both demonstrated moderate predictive ability. Adding the ABI to the clinical model significantly improved discrimination, calibration, and reclassification. Our findings suggest that incorporating ABI into a clinically based model enhances identification of patients with CKD at high risk for PAD who may benefit from early intervention.

Patients with chronic kidney disease (CKD) are at an increased risk for peripheral artery disease (PAD) and its complications.^1^, ^2^, ^3^, ^4^, ^5^ Among US adults aged ≥40, PAD prevalence was 15.3%, 3.8%, and 3.0% in those with estimated glomerular filtration rate (eGFR) <60, 60-90, and >90 mL/min/1.73 m^2^, respectively, based on the 1999-2004 NHANES data.^1^ The Chronic Kidney Disease Prognosis Consortium analyzed 817,084 individuals without PAD at baseline from 21 cohorts. Compared with an eGFR of 95 mL/min/1.73 m^2^, adjusted hazard ratios for incident PAD were 1.22 at eGFR 45 mL/min/1.73 m^2^ and 2.06 at eGFR 15 mL/min/1.73 m^2^. Compared with an albumin-creatinine ratio (ACR) of 5 mg/g, adjusted hazard ratios for PAD were 1.50 at ACR 30 mg/g and 2.28 at ACR 300 mg/g. The adjusted hazard ratio for leg amputation at ACR 300 versus 5 mg/g was 3.68.^3^ Furthermore, patients with both CKD and PAD are at an increased risk for coronary artery disease, heart failure, stroke, and all-cause mortality.^6^, ^7^, ^8^ The American Heart Association recently released a policy statement aiming to reduce nontraumatic lower-extremity amputations by 20% by 2030 through evidence-based PAD prevention and management.^9^^,^^10^ Early PAD risk detection is crucial for prevention and improving outcomes in patients with CKD.

Although the ankle-brachial index (ABI) is widely used to diagnose PAD, the US Preventive Services Task Force found insufficient evidence to evaluate its benefits and harms, citing limited data on its accuracy in identifying asymptomatic individuals who might benefit from PAD or cardiovascular disease (CVD) treatment.^11^ In addition to ABI, several CVD risk factors are significantly associated with the risk of PAD in both the general population and in patients with CKD.^12^^,^^13^ Developing risk prediction models that incorporate both ABI and CVD risk factors is crucial for accurately identifying individuals at high risk for PAD. These models should also be practical for clinical use in PAD risk stratification and prediction. The aim of this study was to develop and validate practical PAD risk prediction models incorporating both CVD risk factors and ABI in patients with CKD.

## Methods

### Study Participants

The Chronic Renal Insufficiency Cohort (CRIC) study is a prospective, longitudinal cohort of 3,939 racially and ethnically diverse adults aged 21-74 with mild-to-moderate CKD (eGFR 20-70 mL/min/1.73 m^2^), enrolled from 7 US centers between May 2003 and August 2008.^14^ Patients with NYHA class 3-4 heart failure, cirrhosis, HIV, polycystic kidney disease, renal cell carcinoma, chronic dialysis, or organ transplant were excluded. After excluding 863 individuals with self-reported PAD or baseline ABI ≤0.9 or >1.4, 3,076 participants were included in the analysis (Figure S1). The CRIC Study was institutional review board-approved at all sites, and all participants provided written informed consent.

### Predictive Variables

Trained and certified staff collected all study data during clinical visits using standard protocols with stringent quality control. Demographics, behavioral risk factors, and medical history were obtained via questionnaire. Three seated blood pressure (BP) measurements were taken with an aneroid sphygmomanometer after ≥5 minutes of rest, following standard procedures.^15^ Pulse pressure was calculated as systolic minus diastolic BP. ABI was measured at baseline and annually after 5 minutes of supine rest, using a continuous-wave Doppler probe in both arms and the posterior tibial and dorsalis pedis arteries. The leg-specific ABI was calculated by dividing the higher systolic BP in the posterior tibial or dorsalis pedis by the higher of the right or left brachial systolic BP.^16^ Blood and urine biomarkers—including total cholesterol, high-density lipoprotein (HDL) cholesterol, low-density lipoprotein (LDL) cholesterol, apolipoprotein A-1, hemoglobin A_1c_, creatinine, cystatin C, urinary albumin-creatinine ratio (UACR), phosphate, alkaline phosphatase, intact parathyroid hormone, hemoglobin, bicarbonate, N-terminal pro b-type natriuretic peptide, high-sensitivity C-reactive protein, and high-sensitivity cardiac troponin T—were measured at a central clinical laboratory at the University of Pennsylvania. eGFR was calculated using the creatinine-based Chronic Kidney Disease Epidemiology Collaboration (CKD-EPI) equation.^17^

### Study Outcome

Study participants were queried about their medical history, including hospitalization and medical procedures, at annual clinical visits and 6-month telephone interviews. PAD information was abstracted from hospital records by a trained research nurse. Clinical PAD events were defined as occlusive artery disease resulting in amputation, peripheral surgical or percutaneous revascularization procedures, any arterial angioplasty, or any artery-to-artery bypass graft. Detailed information regarding revascularization procedures and amputations was extracted, and all medical records of PAD events were adjudicated by ≥2 reviewers. An incident PAD was defined as a new-onset clinical PAD event or an ABI ≤0.9 at a follow-up visit. ABI was measured by trained and certified study personnel using standardized methods at annual clinical visits.

### Statistical Analyses

Log transformations were applied to skewed variables to stabilize variances and normalize distributions. Missing values were imputed using k-nearest neighbor imputation with 5 neighbors.^18^ Nonlinear associations between predictor variables and incident PAD were assessed using spline regression.^19^

#### Model Development

We evaluated 4 risk prediction models: an ABI model that included ABI, age, and sex; a clinical model that included routine clinical variables; a clinical model that included ABI measures in addition to clinical variables; and a biomarker-enhanced model that included additional CVD biomarkers. The association between ABI and incident PAD was U-shaped in the CRIC study participants—those with an ABI of approximately 1.2 had the lowest risk of developing PAD. Therefore, an indicator (ABI <1.2 vs ≥1.2) and its interaction with ABI were included in the models. We evaluated 6 modeling strategies and variable selection procedures to predict the probability of developing PAD within 5 years of baseline: (1) backward elimination Cox proportional hazards regression with a *P* < 0.05 criterion; (2) least absolute shrinkage and selection operator Cox regression with a regularization penalty that maximized cross-validated area under the receiver operating characteristic curve (AUC) (ie, λ = minimum)^20^; (3) boosted trees Cox regression^21^; (4) oblique random survival forests^22^^,^^23^; (5) axis-based random survival forests^24^; and (6) conditional inference random survival forests.

#### Prediction Model Validation

All modeling strategies were internally validated using Monte Carlo split-sampling cross-validation.^25^^,^^26^ Patients were randomly assigned to training and validation sets with a ratio of 80% training to 20% validation. To reduce variability, this process was repeated 100 times, and the validation results were combined to obtain a robust and repeatable estimation of the predictive performance of the models. All modeling approaches performed similarly, so we selected the Cox proportional hazards regression model for its interpretability and ease of clinical use. In the Cox regression models, variables were selected using backward elimination with a criterion of *P* < 0.05. This modeling procedure was applied to all potential predictive variables to develop the final clinical and enriched risk models.

#### Performance Metrics

We evaluated the performance of the developed models using measures of discrimination, calibration, overall goodness of fit, and clinical utility.^27^ Discrimination refers to the ability of a model to correctly identify those who will or will not experience the outcome and was assessed using the time-dependent (ie, 5-year) AUC.^28^ Calibration refers to the agreement between observed outcomes and predictions provided by a given model and was assessed by plotting the observed versus predicted risk across deciles of predicted risk. The scaled Brier score, or index of prediction accuracy (IPA), incorporates information on both discrimination and calibration and was used to evaluate overall goodness of fit.^29^ A higher IPA indicates a better-performing model, an IPA of 1.0 indicates a perfect model, and an IPA ≤0 indicates a useless model. Monte Carlo cross-validation was used to generate 95% confidence intervals (CIs) for the AUCs and IPAs. We used the net reclassification improvement statistic to evaluate the clinical utility of the developed models.^30^ Net reclassification improvement risk categories were based on an estimated risk of 7.5%-20%, corresponding to one of the groups that would benefit from initiation of statin therapy for primary prevention of atherosclerotic vascular diseases.^31^ Bootstrapping with 1,000 replicates was used to generate 95% CIs for the net reclassification improvement.

#### Sensitivity Analysis

We performed a sensitivity analysis to assess model robustness by including end-stage kidney disease (ESKD) as a time-dependent covariate. We also conducted a second sensitivity analysis that used year-0 (baseline) laboratory biomarkers and ABI, combined with year-1 demographic and clinical data, to predict incident PAD between years 1 and 6 in the CRIC study.

Analyses were conducted using SAS version 9.4 (SAS Institute) and R version 4.3.2 (R Project for Statistical Computing). Two-sided *P* values < 0.05 were considered statistically significant for all analyses.

## Results

### Baseline Characteristics

Participants who experienced a PAD event were more likely to be older, female, Black, current smokers, and have a history of CVD or diabetes mellitus. They were also more likely to use antihypertensive, antidiabetic, and statin medications compared with those who did not have a PAD event (Table 1). On average, those who had a PAD event had higher levels of hemoglobin A_1c_, body mass index, systolic BP, pulse pressure, UACR, phosphate, alkaline phosphatase, intact parathyroid hormone, high-sensitivity C-reactive protein, high-sensitivity troponin T, and N-terminal pro b-type natriuretic peptide and lower levels of diastolic BP, eGFR, hemoglobin, and bicarbonate compared with those who did not have an event.

**Table 1:** Baseline Characteristics of CRIC Study Participants

Variables	Overall (n = 3,076)	PAD Events Within 5-Year Follow-Up		
		No (n = 2,564)	Yes (n = 512)	*P*^a^
Age, y	56.6 ± 11.2	56.2 ± 11.3	58.7 ± 10.5	<0.001
Male	1,683 (54.7%)	1,467 (57.2%)	216 (42.2%)	<0.001
Black race	1,245 (40.5%)	976 (38.1%)	269 (52.5%)	<0.001
Current smoking	362 (11.8%)	253 (9.9%)	109 (21.3%)	<0.001
History of CVD	810 (26.3%)	617 (24.1%)	193 (37.7%)	<0.001
Body mass index, kg/m^2^	31.8 ± 7.6	31.6 ± 7.4	33.1 ± 8.2	<0.001
Diabetes mellitus	1,337 (43.5%)	1,046 (40.8%)	291 (56.8%)	<0.001
Hemoglobin A_1c_, %	6.5 ± 1.6	6.5 ± 1.5	6.9 ± 1.8	<0.001
Use of antidiabetic medication	755 (24.7%)	602 (23.6%)	153 (30.2%)	0.002
Systolic BP, mm Hg	127.0 ± 21.7	126.3 ± 21.4	130.6 ± 22.7	<0.001
Diastolic BP, mm Hg	72.4 ± 12.8	72.6 ± 12.7	71.1 ± 13.0	0.02
Pulse pressure, mm Hg	54.7 ± 18.3	53.7 ± 17.9	59.5 ± 19.7	<0.001
Use of BP-lowering medication	2,753 (89.5%)	2,272 (88.6%)	481 (93.9%)	<0.001
Total cholesterol, mg/dL	185.3 ± 45.4	184.6 ± 43.5	188.7 ± 53.4	0.06
LDL cholesterol, mg/dL	104.0 ± 35.7	103.7 ± 34.9	105.6 ± 39.2	0.26
HDL cholesterol, mg/dL	48.1 ± 15.7	48.3 ± 16.0	47.2 ± 14.5	0.14
Apolipoprotein A-1, mg/dL	137.8 ± 30.0	138.2 ± 30.4	136.0 ± 27.5	0.14
Use of statin medications	1,560 (51.1%)	1,255 (49.3%)	305 (60.2%)	<0.001
eGFR, mL/min/1.73 m^2^	43.9 ± 16.1	44.6 ± 16.4	40.2 ± 14.0	<0.001
Urinary ACR, mg/g	45.0 (7.6-407.1)	43.1 (7.0-375.7)	61.3 (10.3-570.7)	0.001
Cystatin C, mg/L	1.5 ± 0.5	1.4 ± 0.5	1.6 ± 0.6	<0.001
Phosphate, mg/dL	3.7 ± 0.7	3.7 ± 0.7	3.8 ± 0.6	<0.001
Alkaline phosphatase, U/L	90.7 ± 34.5	88.8 ± 31.8	100.4 ± 44.2	<0.001
Intact parathyroid hormone, pg/mL	51.1 (33.4-84.3)	50.0 (32.9-82.6)	59.6 (38.6-96.3)	<0.001
Bicarbonate, mmol/L	24.5 ± 3.2	24.6 ± 3.2	24.1 ± 3.3	0.001
Hemoglobin, g/dL	12.7 ± 1.8	12.8 ± 1.8	12.3 ± 1.7	<0.001
hs-CRP, mg/L	2.4 (1.0-6.0)	2.2 (1.0-5.5)	3.6 (1.3-8.2)	<0.001
hs-TnT, pg/mL	10.6 (5.0-21.1)	10.1 (4.8-20.2)	13.6 (6.1-26.1)	<0.001
NT-proBNP, pg/mL	124.0 (55.3-315.8)	114.5 (52.4-284.0)	185.3 (79.9-441.9)	<0.001
Baseline ankle brachial index ≥1.2 to ≤1.4	486 (15.8%)	439 (17.1%)	47 (9.2%)	<0.001

*Note:* Data are presented as n (%), mean ± standard deviation, or median (interquartile range).

Abbreviations: ACR, albumin-creatinine ratio; BP, blood pressure; CRIC, Chronic Renal Insufficiency Cohort; CVD, cardiovascular disease; eGFR, estimated glomerular filtration rate; HDL, high-density lipoprotein; hs-CRP, high-sensitivity C-reactive protein; hs-TnT, high-sensitivity troponin T; LDL, low-density lipoprotein; NT-proBNP, N-terminal pro b-type natriuretic peptide; PAD, peripheral artery disease.

a *P* values are for comparison between participants with PAD and those without PAD.

### Prediction Models

Among the 3,076 participants included in the analyses, 512 developed incident PAD during the first 5 years of follow-up (38.9 events per 1,000 person-years). Among these participants, 41 experienced adjudicated PAD events and 471 developed an ABI ≤0.9. Median follow-up was 5.0 years (mean 4.3 years), during which 95 participants were lost to follow-up and 285 died. Of the deaths, 54 occurred after PAD onset. The remaining 231 deaths were censored, assuming their PAD risk was similar to that of survivors. During follow-up, 485 ESKD events occurred; 64 participants developed PAD before ESKD and 44 developed PAD after ESKD.

The results from the Monte Carlo cross-validation are presented in Table S1. All variable selection procedures yielded similar estimates of AUC and IPA. Due to its interpretability and ease of clinical use, the Cox regression model with a *P* < 0.05 criterion was used to develop final prediction models.

Table 2 shows the hazard ratios with 95% CIs, regression coefficients (β), and information for calculating predicted 5-year risk of PAD for the final models. Model 1 (ABI model) included age, sex, and ABI. Model 2 (CRIC clinical model) included age, sex, current smoking, history of CVD, hemoglobin A_1c_, pulse pressure, LDL cholesterol, HDL-cholesterol, alkaline phosphatase, log (intact parathyroid hormone), bicarbonate, eGFR, and UACR. Model 3 (CRIC clinical model plus ABI) included age, sex, current smoking, history of CVD, hemoglobin A_1c_, pulse pressure, BP-lowering medication, LDL cholesterol, HDL-cholesterol, alkaline phosphatase, hemoglobin, eGFR, UACR, ABI, ABI group (ABI <1.2 vs ≥1.2) and ABI × ABI group. Model 4 (CRIC enriched model) included age, sex, current smoking, history of CVD, hemoglobin A_1c_, pulse pressure, BP-lowering medication, LDL cholesterol, alkaline phosphatase, bicarbonate, eGFR, UACR, ABI, ABI group (ABI <1.2 vs ≥1.2) and ABI × ABI group, log (high-sensitivity C-reactive protein), and log (high-sensitivity troponin T). Example predicted risk calculations using each of the 4 models are available in Tables S2-S5. Predictive models can identify an increased risk of PAD even when the ABI is in the normal range.

**Table 2:** Novel PAD Risk Prediction Models Developed in the CRIC Study

Model Parameters	Model 1: CRIC ABI Only Model		Model 2: CRIC Clinical Model		Model 3: CRIC Clinical Model Plus ABI		Model 4: CRIC Enhanced Model	
	HR (95% CI)	β, per 1-unit	HR (95% CI)	β, per 1-unit	HR (95% CI)	β, per 1-unit	HR (95% CI)	β, per 1-unit
Age, per 5 y	1.12 (1.08-1.16)	0.1110	1.13 (1.08-1.18)	0.1242	1.11 (1.07-1.16)	0.1085	1.1 (1.06-1.15)	0.0967
Male	0.75 (0.64-0.86)	−0.2932	0.62 (0.53-0.72)	−0.4842	0.76 (0.64-0.9)	−0.2757	0.67 (0.57-0.8)	−0.3931
Current smoking	—	—	2.36 (1.95-2.85)	0.8583	2.15 (1.78-2.6)	0.7672	2.11 (1.74-2.55)	0.7458
History of CVD	—	—	1.43 (1.22-1.68)	0.3608	1.38 (1.18-1.62)	0.3238	1.38 (1.17-1.62)	0.3203
Hemoglobin A_1c_, per 0.5%	—	—	1.05 (1.03-1.08)	0.051	1.04 (1.02-1.06)	0.0391	1.04 (1.01-1.06)	0.0357
Pulse pressure, per 10 mm Hg	—	—	1.06 (1.01-1.1)	0.0548	1.05 (1-1.1)	0.0499	1.05 (1.01-1.1)	0.0508
BP-lowering medication	—	—	1.52 (1.11-2.07)	0.4158	1.52 (1.12-2.08)	0.4206	1.47 (1.08-2)	0.383
LDL cholesterol, per 10 mg/dL	—	—	1.04 (1.02-1.06)	0.0415	1.04 (1.02-1.06)	0.0401	1.04 (1.02-1.06)	0.0367
HDL cholesterol, per 10 mg/dL	—	—	0.94 (0.89-0.99)	−0.0636	0.95 (0.9-1)	−0.0556	—	—
Alkaline phosphatase, per 10 U/L	—	—	1.03 (1.01-1.05)	0.0312	1.04 (1.02-1.06)	0.037	1.02 (1-1.05)	0.0235
Log (Intact parathyroid hormone), pg/mL	—	—	1.09 (0.96-1.23)	0.0833	—	—	—	—
Bicarbonate, per 5 mmol/L	—	—	0.87 (0.77-0.98)	−0.1376	—	—	0.88 (0.78-0.99)	−0.1306
eGFR, mL/min/1.73 m^2^	—	—	1.00 (0.99-1.00)	−0.0032	1.00 (0.99-1.00)	−0.0022	1.00 (1.00-1.01)	0.0018
UACR, mg/g	—	—	1.01 (0.97-1.05)	0.0077	1.02 (0.98-1.07)	0.0227	1 (0.96-1.05)	0.005
Hemoglobin, per 5 g/dL	—	—	—	—	0.78 (0.61-1.00)	−0.2497	—	—
Baseline ABI, per 0.05 increase	0.72 (0.68-0.76)	−0.3332	—	—	0.74 (0.7-0.78)	−0.3006	0.74 (0.7-0.78)	−0.298
ABI group	0 (0-0.12)	−7.8294	—	—	0 (0-0.08)	−8.1453	0 (0-0.12)	−7.7968
Baseline ABI × ABI group	1.41 (1.13-1.78)	0.3468	—	—	1.43 (1.14-1.79)	0.3551	1.41 (1.12-1.76)	0.3415
Log (hsCRP), mg/L	—	—	—	—	—	—	1.19 (1.1-1.29)	0.1742
Log (hs-TnT), pg/mL	—	—	—	—	—	—	1.23 (1.11-1.36)	0.2069
**Score calculations**								
Baseline survival at 5 y, $\hat{S}$ (5)	0.8709		0.8481		0.8816		0.8824	
Predicted probability of PAD at 5 y^a^	1 − $\hat{S}$ (5)^exp(∑bX +6.0150)^		1 − $\hat{S}$ (5)^exp(∑bX −2.6361)^		1 − $\hat{S}$ (5)^exp(∑bX +4.1557)^		1-$\hat{S}$ (5)^exp(∑bX+3.4209)^	

Abbreviations: ABI, ankle-brachial index; β, regression coefficient; BP, blood pressure; CI, confidence interval; CRIC, Chronic Renal Insufficiency Cohort; CVD, cardiovascular disease; eGFR, estimated glomerular filtration rate; HDL, high-density lipoprotein; HR, hazard ratio; hs-CRP, high-sensitivity C-reactive protein; hs-TnT, high-sensitivity troponin T; LDL, low-density lipoprotein; NT-proBNP, N-terminal pro b-type natriuretic peptide; PAD, peripheral artery disease; UACR, urinary albumin-creatinine ratio.

a The predicted 5-year probability for each model can be calculated as 1−Ŝ(5)^exp(∑bX − betaavg)^ where b is the regression coefficient (β), X is the individual patient’s level for each risk factor, and betaavg is the sum of the β × value of sample mean.

**Figure 1:**
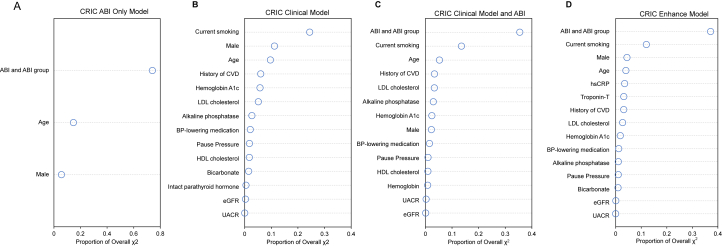
Relative importance of variables in peripheral artery disease risk prediction models for patients with chronic kidney disease patients. Proportion of overall χ^2^ for (A) the ABI model, (B) the CRIC clinical model, (C) the CRIC clinical model plus ABI, and (D) the CRIC biomarker-enhanced model. Higher values for the proportion of overall χ^2^ indicate that the variables are more important relative to others in the model. Abbreviations: ABI, ankle-brachial index; BP, blood pressure; CRIC, Chronic Renal Insufficiency Cohort; CVD, cardiovascular disease; eGFR, estimated glomerular filtration rate; HDL, high-density lipoprotein; hsCRP, high-sensitivity C-reactive protein; LDL, low-density lipoprotein; UACR, urinary albumin-creatinine ratio.

The relative importance of variables included in the 3 models is shown in Fig 1. In model 1, ABI accounted for the greatest proportion of the overall model χ^2^ (Fig 1A). In model 2, current smoking accounted for the greatest proportion of the overall model χ^2^, with the top 3 predictors being smoking, sex, and age (Fig 1B). When ABI variables were included (models 3 and 4), ABI accounted for the greatest proportion of the overall model χ^2^, but current smoking and age remained important (Fig 1C and D).

### Model Performance

**Figure 2:**
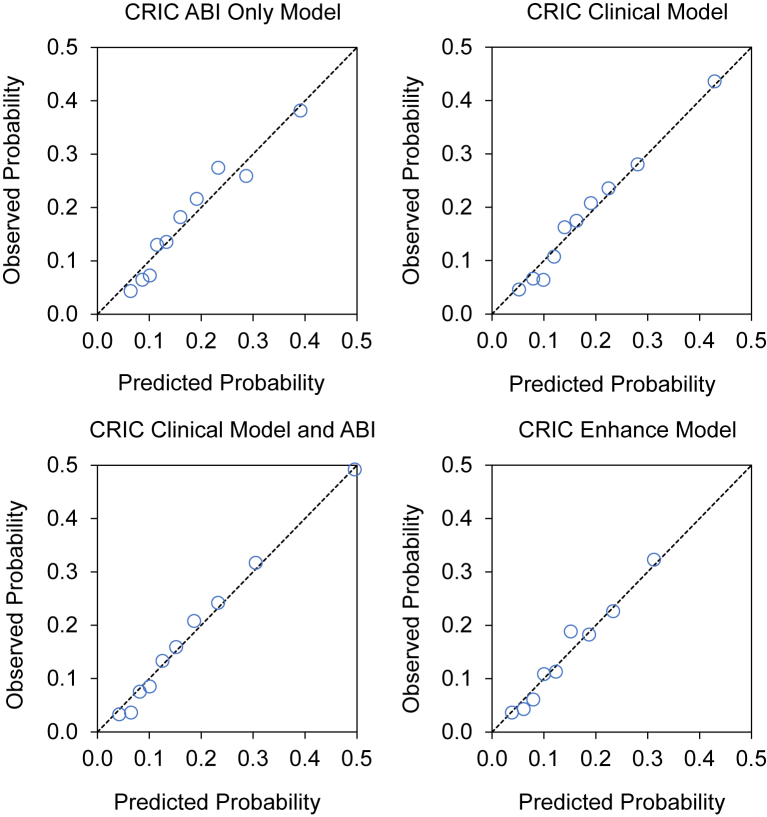
Discrimination and calibration of peripheral artery disease risk prediction models in patients with chronic kidney disease. Results were obtained by aggregating predicted probabilities from Monte Carlo internal cross-validation and then assessing their discrimination (AUC), calibration (predicted probability versus observed events, with each circle representing a decile of predicted probability), and overall goodness of fit (IPA). Observed probability was estimated using the cumulative incidence function. Higher values for AUC (95% CI) and IPA (95% CI) indicate better performing models. Abbreviations: ABI, ankle-brachial index; AUC, area under the receiver operating characteristic curve; CI, confidence interval; CRIC, Chronic Renal Insufficiency Cohort; IPA, index of prediction accuracy.

Performance characteristics of the PAD risk prediction models are summarized in Table 3. The AUC for the ABI model (model 1) was 0.697 (95% CI, 0.688-0.713) and for clinical model (model 2) was 0.682 (95% CI, 0.669-0.691). There was no statistically significant difference between models 1 and 2. After including the ABI variables (model 3), the AUC increased to 0.721 (95% CI, 0.709-0.736), which was significantly higher than that of model 2 (*P* <0 .001). The AUC for the enhanced model (model 4) was 0.724 (95% CI, 0.711-0.745), which did not differ significantly from that of model 3 (*P* = 0.05). Likewise, the IPA was significantly improved from model 2 to model 3 but remained similar between models 3 and 4 (Table 3). All 4 models were well-calibrated across the range of predicted risks. However, models 3 and 4, which included ABI variables, demonstrated better calibration between observed outcomes and predictions compared with model 2, which did not include ABI variables (Fig 2).

**Table 3:** Performance Characteristics of Novel PAD Risk Prediction Models Developed in the CRIC Study

Performance Characteristics	Model 1: CRIC ABI Only Model	Model 2: CRIC Clinical Model	Model 3: CRIC Clinical Model Plus ABI	Model 4: CRIC Enhanced Model
Discrimination				
AUC (95% CI)^a^	0.697 (0.688-0.713)	0.682 (0.669-0.691)	0.721 (0.709-0.736)	0.724 (0.711-0.745)
*P* value comparing successive models^b^	—	0.20	<0.001	0.64
Overall goodness of fit				
IPA (95% CI)^a^	0.062 (0.052-0.071)	0.056 (0.045-0.068)	0.091 (0.076-0.103)	0.093 (0.078-0.108)
*P* value comparing successive models^b^	—	0.09	<0.001	0.05

Abbreviations: ABI, ankle-brachial index; AUC, area under the receiver operating characteristic curve; CI, confidence interval; CRIC, Chronic Renal Insufficiency Cohort; IPA, index of prediction accuracy; PAD, peripheral artery disease.

a Higher values for AUC and IPA indicate better performing models.

b Comparison of model 2 with model 1, and model 3 with model 2.

Compared with the ABI model (model 1), the clinical model (model 2) slightly improved reclassification of non-events (7.4%; 95% CI, 4.4-10.2%). Compared with model 1, the inclusion of ABI variables (model 3) significantly improved reclassification of non-events (19.7%; 95% CI, 17.5-22.0%). In addition, the enhanced model (model 4) significantly improved reclassification of non-events compared with model 1 (22.2%; 95% CI, 19.8-24.5%) and slightly improved reclassification of non-events compared with model 3 (2.5%; 95% CI, 0.8-4.1%). However, the net reclassification improvement of model 4 compared with model 3 was not statistically different when considering the 7.5% and 20% predicted risk thresholds (Table 4).

**Table 4:** Reclassification of Events and Non-Events in the CRIC Study

Model Comparisons^a^	Proportion of Participants Correctly Reclassified^b^		Net Reclassification Index (95% CI)
	Events Reclassified, % (95% CI)	Non-Events Reclassified, % (95% CI)	
Comparing with model 1			
Model 2 versus 1	−1.5 (−7.2 to 4.0)	7.4 (4.4-10.2)	5.9 (−0.8 to 11.6)
Model 3 versus 1	1.3 (−3.2 to 5.9)	19.7 (17.5-22.0)	21.1 (15.9-26.1)
Model 4 versus 1	1.5 (−3.3 to 6.2)	22.2 (19.8-24.5)	23.7 (18.5-28.7)
Comparing with model 2			
Model 3 versus 2	3.3 (−0.7 to 7.8)	11.5 (9.2-14.1)	14.8 (10.1-19.9)
Model 4 versus 2	3.4 (−0.6 to 8.1)	14.1 (11.7-16.9)	17.6 (12.7-22.6)
Comparing with model 3			
Model 4 versus 3	0.4 (−2.3 to 3.0)	2.5 (0.8-4.1)	2.8 (−0.3 to 5.9)

Abbreviations: ABI, ankle-brachial index; CI, confidence interval; CRIC, Chronic Renal Insufficiency Cohort.

a Model 1 indicates the CRIC ABI only model; model 2 indicates CRIC clinical model; model 3 indicates the CRIC model + ABI; and model 4 indicates the CRIC enhanced model.

b Models are compared for reclassification of events to a higher predicted risk category and non-events to a lower predicted risk category, using cut points of 7.5% and 20%.

### Sensitivity Analysis

In the sensitivity analysis including ESKD as a time-dependent covariate, covariate effects remained stable (Table S6). In the analysis using laboratory biomarkers and ABI measured 1 year earlier—combined with current demographic and clinical data—prior-year ABI and biomarkers remained significantly associated with PAD risk, with minimal attenuation in effect sizes (Table S7).

## Discussion

We developed and internally validated prediction models for 5-year PAD risk in patients with CKD. Both the ABI model (age, sex, ABI) and the clinical model (age, sex, smoking, CVD history, hemoglobin A_1c_, pulse pressure, antihypertensive use, LDL cholesterol, HDL cholesterol, alkaline phosphatase, intact parathyroid hormone, bicarbonate, eGFR, UACR) showed moderate predictive ability. Adding ABI to the clinical model significantly improved discrimination, calibration, and reclassification of non-events. The biomarker-enhanced model did not further improve prediction and includes markers not routinely available. These results support using the clinical model with ABI to predict 5-year PAD risk in CKD.

Our findings have important implications for PAD prevention in CKD. PAD is common in CKD and linked to higher CVD and mortality risk. Early detection and intervention may improve outcomes and quality of life.^32^ The US Preventive Services Task Force does not recommend ABI screening for PAD in asymptomatic adults due to limited data on its ability to identify those who could benefit from PAD or CVD treatment.^11^ Additionally, vascular calcification and stiffness can falsely elevate ABI in CKD, leading to pseudo-normal readings. Thus, ABI alone may be less accurate for PAD screening and diagnosis.^33^ Our study suggests that a clinical plus ABI model accurately predicts PAD risk in patients with CKD and could serve as an effective tool to identify high-risk individuals for early intervention.

Currently, ABI is not routinely measured in patients with CKD without PAD history or symptoms. Although Kidney Disease Outcomes Quality Initiative (KDOQI) guidelines recommend PAD screening at dialysis initiation,^34^ the more recent Kidney Disease: Improving Global Outcomes (KDIGO) clinical practice guidelines have not provided guidance on this important issue.^35^ Recently, the 2024 American Heart Association (AHA)/American College of Cardiology (ACC) guidelines recommend resting ABI for PAD screening in high-risk adults aged ≥50 with atherosclerosis risk factors, CKD, or family history, even if they are asymptomatic.^9^ Our study adds important data in this understudied CKD population. ABI is simple, noninvasive, and inexpensive, and may improve PAD risk prediction alongside other CVD risk factors. However, ABI alone may be insufficient, and additional risk factors are needed to assess PAD risk, especially in those patients with normal ABI. Further studies are needed to evaluate these approaches for PAD screening and early prevention of nontraumatic amputation.

The present study has several strengths. We developed and internally validated the first PAD risk prediction model for patients with CKD. All known potential risk factors for PAD were available for model development in the CRIC Study. Baseline asymptomatic PAD, defined by an ABI ≤ 0.9 or ABI > 1.4, was excluded from the analyses to avoid misclassification. PAD events were rigorously adjudicated, and ABI measurements were performed annually with rigorous training and quality control. In addition, the predictive model was internally validated. Furthermore, all predictive variables are either readily available or easily measurable in clinical practice. The 5-year risk prediction model is designed to identify more progressive forms of PAD that may require more aggressive clinical management.

This study has some limitations. First, external validation was limited, as no studies included both PAD outcomes and annual ABI measurements. Some baseline CRIC variables were also unavailable in other cohorts considered for validation. However, we used rigorous internal cross-validation, similar to the method for developing the ACC/AHA atherosclerotic CVD risk equations.^36^ Second, the toe-brachial index was not measured. However, current guidelines recommend toe-brachial index only for individuals with ABI >1.4 and clinical suspicion of PAD.^9^ In our study, participants with baseline ABI >1.4 were excluded. Furthermore, the risk prediction models should be externally validated in diverse CKD populations or clinical trials to assess their utility in guiding prevention.

## Conclusions

In patients with CKD, a prediction model that combined routinely available clinical variables with ABI outperformed models using either clinical variables or ABI alone, underscoring the added value of routine ABI measurement for PAD screening and risk prediction, as recommended by the 2024 AHA/ACC guidelines. Adding non-routinely measured CVD biomarkers did not further improve predictive performance. The clinical prediction model with ABI could identify CKD patients at high PAD risk who may benefit from early preventive interventions. Future studies should validate the model in large, diverse CKD populations and assess its utility in PAD screening and prevention trials.
