# Supplementary material for: Five-Year Risk Prediction Models for Peripheral Artery Disease in Patients With Chronic Kidney Disease
Source: Kidney Med. 2026 Apr 3;8(6):101351. doi: 10.1016/j.xkme.2026.101351 (PMC13156743; doi:10.1016/j.xkme.2026.101351)
Supplement: Supplementary File (PDF). Figure S1 and Table S1-S7 [file mmc1.pdf]

Supplementary Material

Figure S1. Participant Flowchart

Table S1. Monte Carlo Internal Validation of 5-year Risk Prediction of Peripheral Artery Disease in the CRIC Study

Table 2. Calculation of Predicted 5-year Risk of PAD among Patients with Chronic Kidney Disease (ABI Only Model)

Table S3. Calculation of Predicted 5-year Risk of PAD among Patients with Chronic Kidney Disease (Clinical Model)

Table S4. Calculation of Predicted 5-year Risk of PAD among Patients with Chronic Kidney Disease (Clinical Model + ABI)

Table S5. Calculation of Predicted 5-year Risk of PAD among Patients with Chronic Kidney Disease (Enhance Model)

Table S6. Five-year Risk Prediction Models for PAD Including Time-dependent ESKD among Patients with Chronic Kidney Disease

Table S7. Five-year PAD Risk Prediction Models using Biomarkers and ABI Measured One Year Earlier among Patients with Chronic Kidney Disease

Supplemental Figure 1. Participant Flowchart

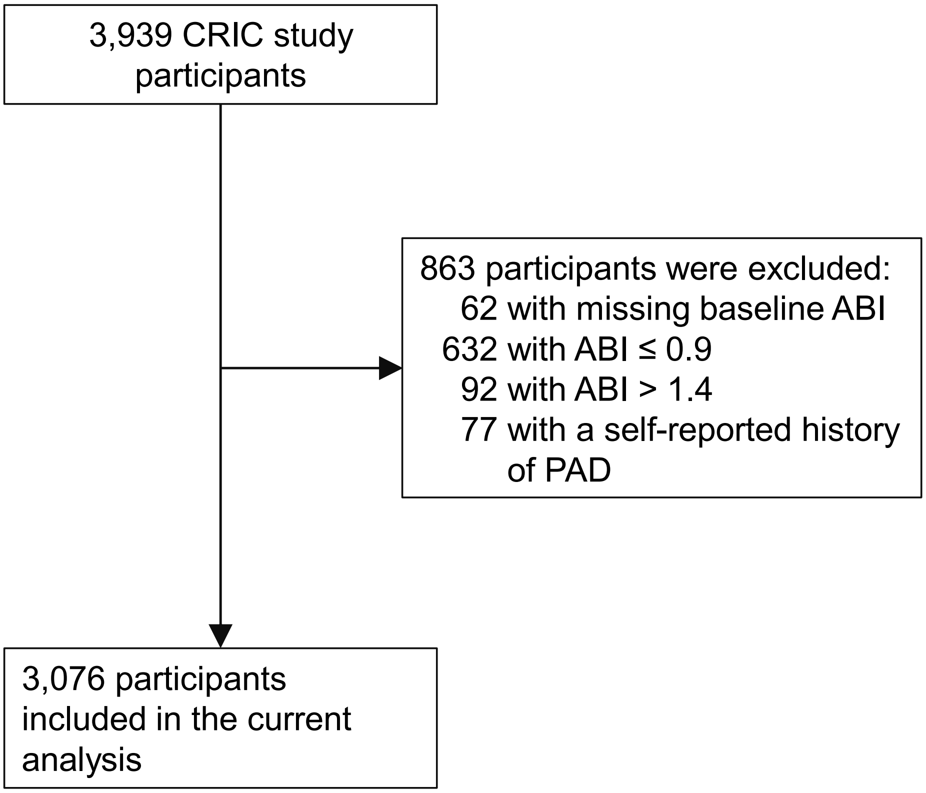

Table S1. Monte Carlo Internal Validation of 5-year Risk Prediction of Peripheral Artery Disease in the CRIC Study

Candidate Variables and Selection Procedures	ABI Only Model		Clinical Model		Clinical Model plus ABI		Enhance Model	
	AUC (95% CI) *	IPA (95% CI) *	AUC (95% CI) *	IPA (95% CI) *	AUC (95% CI) *	IPA (95% CI) *	AUC (95% CI) *	IPA (95% CI) *
Oblique Random Survival Forest	0.693 (0.683, 0.702)	0.059 (0.050, 0.067)	0.682 (0.671, 0.696)	0.053 (0.041, 0.068)	0.730 (0.713, 0.743)	0.097 (0.084, 0.113)	0.731 (0.718, 0.745)	0.098 (0.088, 0.108)
Axis-based Random Survival Forest	0.683 (0.670, 0.692)	0.056 (0.048, 0.060)	0.669 (0.654, 0.680)	0.046 (0.037, 0.055)	0.712 (0.697, 0.725)	0.080 (0.070, 0.090)	0.723 (0.709, 0.741)	0.086 (0.071, 0.098)
Conditional inference Random Survival Forest	0.677 (0.666, 0.686)	0.051 (0.041, 0.058)	0.673 (0.661, 0.685)	0.048 (0.036, 0.060)	0.725 (0.708, 0.738)	0.092 (0.080, 0.106)	0.729 (0.718, 0.742)	0.094 (0.082, 0.107)
Backward Cox Regression †	0.697 (0.688, 0.713)	0.062 (0.052, 0.071)	0.682 (0.669, 0.691)	0.056 (0.045, 0.068)	0.721 (0.709, 0.736)	0.091 (0.076, 0.103)	0.724 (0.711, 0.745)	0.093 (0.078, 0.108)
LASSO Cox Regression	0.697 (0.688, 0.715)	0.062 (0.053, 0.071)	0.683 (0.673, 0.697)	0.046 (0.040, 0.052)	0.720 (0.705, 0.738)	0.075 (0.064, 0.083)	0.723 (0.703, 0.742)	0.070 (0.060, 0.077)
Boosted trees Cox Regression	0.687 (0.672, 0.696)	0.043 (0.038, 0.049)	0.676 (0.661, 0.694)	0.033 (0.024, 0.042)	0.724 (0.705, 0.740)	0.074 (0.064, 0.083)	0.728 (0.713, 0.741)	0.072 (0.066, 0.079)

Abbreviations: AUC = area under the ROC curve; CI = confidence interval; IPA = index of prediction accuracy; LASSO = least absolute shrinkage and selection operator.

* AUC and IPA point and interval estimates were obtained using Monte Carlo cross-validation. Higher values for the AUC and IPA indicate better performing models.

† Models were fitted using Cox proportional hazards regression and variables selected using backward elimination with a criterion of $P<0.05$.

Table S2. Calculation of Predicted 5-year Risk of PAD among Patients with Chronic Kidney Disease (ABI Only Model)

Variable	Coefficient	Example Participant 1		Example Participant 2		Example Participant 3	
		Patient Values	Coefficient × Values	Patient Values	Coefficient × Values	Patient Values	Coefficient × Values
Age (years)	0.0222	40	0.8884	55	1.2216	73	1.6213
Sex (1 if male)	-0.2932	0	0.0000	1	-0.2932	0	0.0000
ABI	-6.664	1.0	-6.6640	1.1	-7.3304	1.2	-7.9968
ABI group (1 if ABI ≥1.2 to ≤1.4, 0 if ABI >0.9 to <1.2)	-7.8294	0	0.0000	0	0.0000	1	-7.8290
Low ABI × ABI group	6.936	0	0.0000	0	0.0000	1.2	8.3232
**Risk Calculation**							
Sum		-5.7756		-6.4021		-5.8813	
Probability (PAD event)		0.1611		0.0896		0.1462	
5-year risk, % *		16.11		8.96		14.62	

Abbreviations: ABI = ankle-brachial index; PAD = peripheral artery diseases.

* The 5-year risk can be calculated as $1-0.8709^{\exp(\Sigma bX+6.0150)}$ where b is the regression coefficient (beta), and X is the level for each risk factor.

Table S3. Calculation of Predicted 5-year Risk of PAD among Patients with Chronic Kidney Disease (Clinical Model)

Variable	Coefficient	Example Participant 1		Example Participant 2		Example Participant 3	
		Patient Values	Coefficient × Values	Patient Values	Coefficient × Values	Patient Values	Coefficient × Values
Age (years)	0.0248	40	0.992	55	1.364	73	1.8104
Sex (1 if male)	-0.4842	0	0	1	-0.4842	0	0
Current smoking (1 if yes)	0.8583	0	0	0	0	0	0
History of CVD (1 if yes)	0.3608	0	0	0	0	1	0.3608
Hemoglobin A1c (%)	0.102	5.2	0.5304	5.6	0.5712	7.5	0.765
Pulse pressure (mm Hg)	0.0055	40	0.22	55	0.3025	55	0.3025
BP-lowering medication (1 if yes)	0.4158	1	0.4158	1	0.4158	1	0.4158
LDL cholesterol (mg/dL)	0.0042	146	0.6132	81	0.3402	83	0.3486
HDL cholesterol (mg/dL)	-0.0064	51	-0.3264	50	-0.32	46	-0.2944
Alkaline phosphatase (U/L)	0.0031	72	0.2232	100	0.31	100	0.31
Intact parathyroid hormone (pg/mL) *	0.0833	ln(40+1)	0.3093	ln(100+1)	0.3844	ln(100+1)	0.3844
Bicarbonate (mmol/L)	-0.0275	26	-0.715	26	-0.715	22	-0.605
eGFR (mL/min/1.73 m2)	-0.0032	31.0	-0.0992	45.7	-0.1462	29.3	-0.0938
UACR (mg/g)	0.0077	2.4	0.0185	3.1	0.0239	3.5	0.027
**Risk Calculation**							
Sum		2.1818		2.0466		3.7313	
Probability (PAD event)		0.0993		0.0873		0.389	
5-year risk, % †		9.93		8.73		38.9	

Abbreviations: ABI = ankle-brachial index; BP = blood pressure; CRIC = Chronic Renal Insufficiency Cohort; CVD = cardiovascular disease; HDL = high-density lipoproteins; LDL = low-density lipoprotein; PAD = peripheral artery diseases.

* Patient values are natural log transformed. For example, if a patient has a value of hsCRP as 1.2, then the transformation will be ln(1.2+1);

† The 5-year risk can be calculated as $1\text{-}0.8481^{\exp(\sum bX-2.6361)}$ where b is the regression coefficient (beta), and X is the level for each risk factor.

Table S4. Calculation of Predicted 5-year Risk of PAD among Patients with Chronic Kidney Disease (Clinical Model + ABI)

Variable	Coefficient	Example Participant 1		Example Participant 2		Example Participant 3	
		Patient Values	Coefficient × Values	Patient Values	Coefficient × Values	Patient Values	Coefficient × Values
Age (years)	0.021698	40	0.8679	55	1.1934	73	1.584
Sex (1 if male)	-0.27572	0	0	1	-0.2757	0	0
Current smoking (1 if yes)	0.767164	0	0	0	0	0	0
History of CVD (1 if yes)	0.323835	0	0	0	0	1	0.3238
Hemoglobin A1c (%)	0.078204	5.2	0.4067	5.6	0.4379	7.5	0.5865
Pulse pressure (mm Hg)	0.004988	40.0	0.1995	55	0.2743	55	0.2743
BP-lowering medication (1 if yes)	0.420621	1	0.4206	1	0.4206	1	0.4206
LDL cholesterol (mg/dL)	0.004011	146	0.5856	81	0.3249	146	0.5856
HDL cholesterol (mg/dL)	-0.00556	51	-0.2836	50	-0.278	46	-0.2558
Alkaline phosphatase (U/L)	0.003699	72	0.2663	140	0.5179	140	0.5179
eGFR (mL/min/1.73 m2)	-0.00219	31.0	-0.0679	45.7	-0.1001	29.3	-0.0642
UACR (mg/g)	0.02275	2.4	0.0546	3.1	0.0705	3.5	0.0796
Hemoglobin (g/dL)	-0.04994	12.8	-0.6392	14.9	-0.7441	12.4	-0.6193
ABI	-6.01228	1.0	-6.0123	1.1	-6.6135	1.2	-7.2147
ABI group (1 if ABI ≥1.2 to ≤1.4, 0 if ABI >0.9 to <1.2)	-8.14535	0	0	0	0	1	-8.1454
Low ABI × ABI group	7.101749	0	0	0	0	1.2	8.5221
**Risk Calculation**							
Sum		-4.2017		-4.7718		-3.4048	
Probability (PAD event)		0.1134		0.0658		0.2343	
5-year risk, % *		11.34		6.58		23.43	

Abbreviations: ABI = ankle brachial index; BP = blood pressure; CRIC = Chronic Renal Insufficiency Cohort; CVD = cardiovascular disease; HDL = high-density lipoproteins; LDL = low-density lipoprotein; PAD = peripheral artery diseases.

* The 5-year risk can be calculated as $1\text{-}0.8816^{\exp(\sum bX+4.1557)}$ where b is the regression coefficient (beta), and X is the level for each risk factor.

Table S5. Calculation of Predicted 5-year Risk of PAD among Patients with Chronic Kidney Disease (Enhance Model)

Variable	Coefficient	Example Participant 1		Example Participant 2		Example Participant 3	
		Patient Values	Coefficient × Values	Patient Values	Coefficient × Values	Patient Values	Coefficient × Values
Age (years)	0.019338	40	0.7735	55	1.0636	73	1.4117
Sex (1 if male)	-0.39313	0	0	1	-0.3931	0	0
Current smoking (1 if yes)	0.745833	0	0	0	0	0	0
History of CVD (1 if yes)	0.320312	0	0	0	0	1	0.3203
Hemoglobin A1c (g/dL)	0.071443	5.2	0.3715	5.6	0.4001	7.5	0.5358
Pause pressure (mm Hg)	0.005079	40.0	0.2032	50	0.254	55	0.2793
BP-lowering medication (1 if yes)	0.382983	1	0.383	1	0.383	1	0.383
LDL cholesterol (mg/dL)	0.003669	146	0.5357	81	0.2972	146	0.5357
Alkaline phosphatase (U/L)	0.00235	64	0.1504	140	0.329	140	0.329
Bicarbonate (mmol/L)	-0.02613	26	-0.6794	26	-0.6794	22	-0.5749
eGFR (mL/min/1.73 m2)	0.001808	31.0	0.056	45.7	0.0826	29.3	0.053
UACR (mg/g)	0.004981	2.4	0.012	3.1	0.0154	3.5	0.0174
ABI	-5.95942	1.0	-5.9594	1.1	-6.5554	1.2	-7.1513
ABI group (1 if ABI ≥1.2 to ≤1.4, 0 if ABI >0.9 to <1.2)	-7.79675	0	0	0	0	1	-7.7968
Low ABI × ABI group	6.830953	0	0	0	0	1.2	8.1971
hsCRP mg/L *	0.17422	ln(1.2+1)	0.1374	ln(3.0+1)	0.2415	ln(6.0+1)	0.339
Troponin-T (pg/mL) *	0.206889	ln(5+1)	0.3707	ln(12+1)	0.5307	ln(21+1)	0.6395
**Risk Calculation**							
Sum		-3.6455		-4.0308		-2.482	
Probability (PAD event)		0.0951		0.0657		0.2738	
5-year risk, % *		9.51		6.57		27.38	

Abbreviations: ABI = ankle brachial index; BP = blood pressure; CRIC = Chronic Renal Insufficiency Cohort; CVD = cardiovascular disease; hsCRP = high-sensitive C-reactive protein; LDL = low-density lipoprotein; PAD = peripheral artery diseases; Troponin-T = high sensitive troponin T.

* Patient values are natural log transformed. For example, if a patient has a value of hsCRP as 1.2, then the transformation will be ln(1.2+1).

† The 5-year risk can be calculated as $1-0.8824^{\exp(\sum bX+3.4209)}$ where b is the regression coefficient (beta), and X is the level for each risk factor.

Table S6. Five-year Risk Prediction Models for PAD Including Time-dependent ESKD among Patients with Chronic Kidney Disease

Model Parameters	Model 1: CRIC ABI Only Model		Model 2: CRIC Clinical Model		Model 3: CRIC Clinical Model Plus ABI		Model 4: CRIC Enhance Model	
	HR (95% CI)	β, per 1-unit	HR (95% CI)	β, per 1-unit	HR (95% CI)	β, per 1-unit	HR (95% CI)	β, per 1-unit
ESKD	1.62 (1.3, 2.02)	0.4842	1.08 (0.84, 1.39)	0.0754	1.12 (0.87, 1.44)	0.1103	1.14 (0.88, 1.46)	0.1288
Age, per 5 years	1.13 (1.09, 1.17)	0.1198	1.13 (1.09, 1.18)	0.1256	1.12 (1.07, 1.17)	0.1105	1.1 (1.06, 1.15)	0.0993
Male sex	0.74 (0.64, 0.85)	-0.3054	0.61 (0.53, 0.72)	-0.4866	0.76 (0.64, 0.89)	-0.2797	0.67 (0.57, 0.8)	-0.3944
Current smoking			2.36 (1.96, 2.85)	0.8592	2.15 (1.78, 2.6)	0.7663	2.11 (1.74, 2.55)	0.7448
History of CVD			1.44 (1.22, 1.68)	0.3612	1.38 (1.18, 1.62)	0.3246	1.38 (1.17, 1.62)	0.3213
Hemoglobin A1c, per 0.5%			1.05 (1.03, 1.08)	0.0508	1.04 (1.02, 1.06)	0.0392	1.04 (1.01, 1.06)	0.0358
Pulse pressure, per 10 mm Hg			1.06 (1.01, 1.1)	0.0538	1.05 (1, 1.1)	0.0487	1.05 (1, 1.1)	0.0493
BP lowering medication			1.52 (1.11, 2.07)	0.4176	1.53 (1.12, 2.08)	0.4229	1.47 (1.08, 2)	0.3855
LDL cholesterol, per 10 mg/dL			1.04 (1.02, 1.06)	0.0416	1.04 (1.02, 1.06)	0.0402	1.04 (1.02, 1.06)	0.0369
HDL cholesterol, per 10 mg/dL			0.94 (0.89, 0.99)	-0.0637	0.95 (0.9, 1)	-0.0556		
Alkaline phosphatase, per 10 U/L			1.03 (1.01, 1.05)	0.0312	1.04 (1.02, 1.06)	0.0369	1.02 (1, 1.04)	0.0233
Log (Intact parathyroid hormone), pg/mL			1.08 (0.95, 1.23)	0.0803				
Bicarbonate, per 5 mmol/L			0.87 (0.77,	-0.1367			0.88 (0.78,	-0.1292

			0.99)				0.99)	
eGFR, mL/min/1.73 m2			1 (0.99, 1)	-0.0029	1 (0.99, 1)	-0.0017	1 (1, 1.01)	0.0024
UACR, mg/g			1 (0.96, 1.05)	0.0046	1.02 (0.98, 1.06)	0.0179	1 (0.96, 1.04)	-0.0005
Hemoglobin, per 5 g/dL					0.78 (0.61, 1.01)	-0.2425		
Baseline ABI, per 0.05 increase	0.72 (0.68, 0.76)	-0.3305			0.74 (0.7, 0.78)	-0.3007	0.74 (0.7, 0.78)	-0.2982
ABI group	0 (0, 0.1)	-8.0331			0 (0, 0.08)	-8.1868	0 (0, 0.11)	-7.8389
Baseline ABI × ABI group	1.42 (1.13, 1.79)	0.3538			1.43 (1.14, 1.79)	0.3566	1.41 (1.13, 1.77)	0.3431
Log (hsCRP), mg/L							1.19 (1.1, 1.3)	0.1766
Log (Troponin-T), pg/mL							1.23 (1.11, 1.36)	0.2047
**Score calculations**								
Baseline survival at 5 years, $\hat{S}(5)$	0.8681		0.8437		0.8795		0.8806	
Predicted probability of PAD at 5 years [*]	$1-\hat{S}(5)^{\exp(\sum bX+5.7974)}$		$1-\hat{S}(5)^{\exp(\sum bX-2.6740)}$		$1-\hat{S}(5)^{\exp(\sum bX+4.0848)}$		$1-\hat{S}(5)^{\exp(\sum bX+3.3596)}$	

Abbreviations: ABI = ankle brachial index; β = regression coefficient; BP = blood pressure; CRIC = Chronic Renal Insufficiency Cohort; CVD = cardiovascular disease; eGFR=estimated glomerular filtration rate; HDL = high-density lipoproteins; hsCRP = high-sensitive C-reactive protein; HR = hazard ratio; LDL = low-density lipoprotein; NT-proBNP = N-terminal pro b-type natriuretic peptide; PAD = peripheral artery diseases; Troponin-T = high sensitive troponin T; UACR=urine albumin creatinine rate.

[*] The predicted 5-year probability for each model can be calculated as $1-S(5)^{\exp(\sum bX-betaavg)}$ where b is the regression coefficient (β), X is the individual patient's level for each risk factor, and betaavg is the sum of the $\beta\times$value of sample mean.

Table S7. Five-year PAD Risk Prediction Models using Biomarkers and ABI Measured One Year Earlier among Patients with Chronic Kidney Disease

**Model Parameters**	**Model 1: CRIC ABI Only Model**		**Model 2: CRIC Clinical Model**		**Model 3: CRIC Clinical Model Plus ABI**		**Model 4: CRIC Enhance Model**	
	**HR (95% CI)**	**β, per 1-unit**	**HR (95% CI)**	**β, per 1-unit**	**HR (95% CI)**	**β, per 1-unit**	**HR (95% CI)**	**β, per 1-unit**
Age, per 5 years	1.13 (1.08, 1.18)	0.1219	1.16 (1.11, 1.22)	0.1513	1.15 (1.1, 1.21)	0.1399	1.13 (1.08, 1.19)	0.1245
Male sex	0.7 (0.58, 0.83)	-0.3623	0.62 (0.51, 0.75)	-0.4781	0.79 (0.65, 0.96)	-0.2374	0.64 (0.53, 0.78)	-0.4426
Current smoking			2.37 (1.87, 3.01)	0.864	2.29 (1.8, 2.9)	0.8274	2.23 (1.76, 2.83)	0.803
History of CVD			1.55 (1.28, 1.87)	0.4364	1.5 (1.24, 1.82)	0.4074	1.48 (1.22, 1.79)	0.393
Hemoglobin A1c, per 0.5%			1.04 (1.02, 1.08)	0.0439	1.03 (1, 1.06)	0.033	1.03 (1, 1.06)	0.0296
Pulse pressure, per 10 mm Hg			1.07 (0.99, 1.16)	0.0704	1.06 (0.98, 1.15)	0.0617	1.05 (0.97, 1.14)	0.0529
BP lowering medication			1.5 (1.05, 2.14)	0.4042	1.49 (1.04, 2.12)	0.3965	1.44 (1.01, 2.06)	0.3658
LDL cholesterol, per 10 mg/dL			1.05 (1.02, 1.07)	0.0466	1.05 (1.03, 1.08)	0.0495	1.05 (1.02, 1.07)	0.0445
HDL cholesterol, per 10 mg/dL			0.98 (0.92, 1.04)	-0.021	0.99 (0.93, 1.05)	-0.0124		
Alkaline phosphatase, per 10 U/L			1.04 (1.01, 1.07)	0.04	1.05 (1.02, 1.07)	0.0454	1.04 (1.01, 1.06)	0.0373
Log (Intact parathyroid hormone), pg/mL			1.12 (0.96, 1.31)	0.117				
Bicarbonate, per 5 mmol/L			0.93 (0.8, 1.08)	-0.0758			0.94 (0.81, 1.09)	-0.0582
eGFR, mL/min/1.73 m2			1 (0.99, 1.01)	-0.0019	1 (0.99, 1.01)	-0.0005	1 (0.99, 1.01)	0.0012
UACR, mg/g			1.03 (0.98, 1.08)	0.0274	1.04 (0.99, 1.09)	0.0369	1.03 (0.98, 1.08)	0.0266

Hemoglobin, per 5 g/dL					0.62 (0.46, 0.83)	-0.4846		
Baseline ABI, per 0.05 increase	0.75 (0.71, 0.8)	-0.2863			0.77 (0.73, 0.82)	-0.2553	0.78 (0.73, 0.83)	-0.2522
ABI group	0 (0, 0.39)	-7.6032			0 (0, 0.13)	-8.68	0 (0, 0.13)	-8.6603
Baseline ABI × ABI group	1.39 (1.07, 1.81)	0.3309			1.45 (1.11, 1.88)	0.3687	1.45 (1.11, 1.88)	0.3686
Log (hsCRP), mg/L							1.17 (1.06, 1.3)	0.1573
Log (Troponin-T), pg/mL							1.22 (1.08, 1.38)	0.2005
**Score calculations**								
Baseline survival at 6 years, $\hat{S}(5)$	0.8967		0.8815		0.9082		0.9090	
Predicted probability of PAD at 6 years *	$1-\hat{S}(5)^{\exp(\sum bX+4.9333)}$		$1-\hat{S}(5)^{\exp(\sum bX-3.9593)}$		$1-\hat{S}(5)^{\exp(\sum bX+2.8559)}$		$1-\hat{S}(5)^{\exp(\sum bX+1.6023)}$	

Abbreviations: ABI = ankle brachial index; β = regression coefficient; BP = blood pressure; CRIC = Chronic Renal Insufficiency Cohort; CVD = cardiovascular disease; eGFR=estimated glomerular filtration rate; HDL = high-density lipoproteins; hsCRP = high-sensitive C-reactive protein; HR = hazard ratio; LDL = low-density lipoprotein; NT-proBNP = N-terminal pro b-type natriuretic peptide; PAD = peripheral artery diseases; Troponin-T = high sensitive troponin T; UACR=urine albumin creatinine rate.

* The predicted 5-year probability for each model can be calculated as $1-S(5)^{\exp(\sum bX-betaavg)}$ where b is the regression coefficient (*β*), X is the individual patient's level for each risk factor, and betaavg is the sum of the *β*×value of sample mean.
